# Etiopathogenesis and prognostic implications of autoimmune hemolytic anemia association with chronic lymphocytic leukemia B

**Published:** 2012

**Authors:** MM Oprea, AM Ivanescu, A Colita, E Coles, RC Momanu, N Berbec, AR Lupu

**Affiliations:** * Hematology Department, Coltea Clinical Hospital, Bucharest; ** “Carol Davila” University of Medicine and Pharmacy, Bucharest; *** Roche Romania, Bucharest

**Keywords:** autoimmune hemolytic anemia, chronic lymphocytic leukemia B, autoreactive B-lymphocytes, self-reactive T cells, AHAI - autoimmune hemolytic anemia, CLL - chronic lymphocytic leukemia, TH - helper T cells, Treg - regulatory T cells, Fas /Fas ligand - apoptosis pathway that causes cell death, RBC - red blood cells, IgG - immunoglobulin G CD cells, DNA - deoxyribonucleic acid, APC - antigen presentation cells, Rh protein - Rhesus - type of protein on the surface of red blood cells, ALPS - autoimmune/ lymphoproliferative syndrome, ALD - autoimmune lymphoproliferative disease, BCR - B cell receptor, IgVH - immunoglobulin heavy chain, RNA - Ribonucleic acid, ZAP70 - zeta-chain associated protein kinase 70kDa

## Abstract

Biological complexity of mechanisms of autoimmune hemolytic anemia (AHAI) in chronic lymphocytic leukemia B (CLL) and the relation cause / effect between these two diseases has been extensively researched, but currently is still far from being completely understood.

It is known that the immune system has an important role in the pathogenesis of autoimmune diseases but also in the chronic lymphoproliferative malignancies. In this process of autoimmunity associated with immunodeficiency, the CLL neoplastic cells, the non-malignant B cells, T cells, and the cellular microenvironment cells are also involved. CLL pathological lymphocytes change peripheral immune tolerance acting as an antigen presenting cells and / or cells expressing inhibitory cytokines. The two subpopulations of T cells also have an important place: self-reactive T helper cells (TH) and regulatory T cells (Treg). The Fas / Fas ligand - cell death mechanism has a significant role both in maintaining cellular homeostasis, malignant hematopoietic cell expansion and the development of autoimmune disorders, including AIHA.

The article reviews the etiopathogenesis of the autoimmune mechanism of AIHA in CLL, and its impact on the prognosis and long – term survival of patients with chronic lymphocytic leukemia B.

## Introduction

Chronic lymphocytic leukemia B (CLL) is a chronic lymphoproliferative syndrome characterized by clonal proliferation and accumulation of morphologically mature lymphocytes, but with immature function [**1**]. 5-10% of autoimmune disorders associated with CLL are autoimmune cytopenias. Of these, the most common complication (50- 60%) is autoimmune hemolytic anemia (AIHA) [**2,3**]. AIHA is an autoimmune disease in which red blood cells (RBC) are destroyed prematurely due to production of antierythrocyte autoantibodies. The most common form of AIHA is characterized by the presence of “warm”-type autoantibodies, which are immunoglobulin G (IgG) type and react optimally at 37o Celsius, causing extravascular RBC destruction by tissue macrophages [**4**].

AIHA may occur alone or in combination with other autoimmune disorders like thrombocytopenia (Evans syndrome), and less frequent: pure red blood cell aplasia or autoimmune granulocytopenia, AIHA may precede by months / years the onset of CLL, can be detected at diagnosis or may occur as a complication of the disease [**2,5**]. Regarding the incidence of AIHA, no statistical differences was observed concerning the time to cytopenia onset during CLL course (before diagnosis, at diagnosis or in evolution), treatment modality (fludarabine - 4% vs. alkylation agents - 5%), and / or long-term survival (median survival: 8-9 years) [**6**].

### The etiopathogenic mechanism of autoimmune hemolytic anemia in CLL

The immune system has an important role in autoimmunity - infection / inflammation and development of malignant lymphomas, but the exact relationship between cause and effect is not yet known [**7**]. An immune response is essential for protection against cancer development, but immune activation can lead to loss of immune tolerance and induction of autoimmunity. Two assumptions can be made about the pathogenesis of antibodies production CLL associated AIHA : the abnormal function of the immune system can cause the formation of antibodies along with cancer cells proliferation, or that tumor clone itself causes the immune system [**8**].

Studies investigating the pathogenic mechanisms of CLL associated AIHA are focused on malignant mature CD5 + B cells [9]. Immunodeficiency associated with active CLL or high stage disease may favor production of anti-red blood cells (RBC) antibodies by the CD5 + leukemic clone. Most patients with CLL show clinical signs of active disease when AIHA is diagnosed. Response was observed after treatment of both diseases, the clinical course indicating that there is a strong link between disease activity and AIHA. This is explained by biological correlation between CLL and self-reactivity, CD5 + B-cells being involved in both [**10**].

The biological explanation for the association between AIHA and CLL is complex and not fully understood [**8,11**]. Autoreactive lymphocytes could be the result of the gene segment rearrangement in the DNA of precursor lymphocytes. In most individuals, the intact cell-death mechanisms could inactivate and/or delete these autoreactive clones, but in people with a certain predisposition, a few of these cells continue to proliferate slowly in peripheral lymphoid organs. In addition, the alteration of mechanisms that prevent the expansion of autoreactive cells can cause several autoimmune manifestations [**9**].

Is not unknown that patients with immune system tumors, like CLL, are at risk of develop autoantibodies against hematopoietic cells [**9,12**]. In the blood of these patients two types of antibodies were identified: immunoglobulins secreted directly by CLL cells, and polyclonal RBC-reactive autoantibodies, with a different specificity and isotype from the first one [**13**]. The cause of anti-RBC polyclonal IgG production in CLL patients is not well known; the alteration of the immune tolerance could be determined by the nonmalignant B cells, abnormal antigen presentation by CLL cells, and the T cell dysfunction [**2**].

a) The studies have shown that the pathogenic autoantibodies could be produced by the residual nonmalignant B cells [**1,13**]. In healthy persons, autoreactive B and T cells (part of a normal immune repertoire) stay dormant, but if they are activated, could be capable to produce autoimmune disease [**5,14**]. In the context of pathogenic autoantibody production, the B cell is largely studied, it has the ability to process and present antigen, also to express the costimulatory molecules necessary to stimulate TH cells, in a conventional immune response [**13,14**]. The role of B cells in presenting autoantigen has been shown [**13,15**] in murine models of autoimmune diseases, such as diabetes [**16**] and multiple sclerosis [**17**].

b) The role of CD5+ CLL B cells in autoantigen presentation is complex and unclear. In vitro, the malignant cells appear to be inefficient like antigen presentation cells (APC). This possibility should not be disregarded, additional reports have shown that autoimmune diseases, including AIHA, are initiated by changes in autoantigen presentation such as the recruitment of new APC types [**13,14**], and also by activation of costimulatory molecules such as CD80; the antigen presentation is enhanced after meeting with T cells expressing CD40 ligand [**13,15**]. Although it is known that CLL cells have reduced role as antigen presenting cells for conventional antigens, they are highly effective in stimulating the proliferation of TH cells specific for Rh protein [**13**]. Murine studies have demonstrated that IgG autoantibodies against RBC proteins are dependent on the activation of autoreactive TH cells specific for these antigens, an Rh-reactive helper response has previously been identified in human patients with primary AIHA, and in patients with AIHA secondary to CLL [**13,18**].

c) Also, frequently involved in producing AIHA is the richest membrane protein in the red blood cells, the anion exchanger known as band 3 (B3). Because CLL cells bind with high avidity to B3 and produce in vitro phagocytosis of this protein, it is thought that the malignant lymphocytic cells present epitopes B3 to self-reactive T cell [**2,19**].

d) On the other hand, studies have shown that both autoreactive T helper cells (TH) and regulatory T cells (Treg) play an important role for induction of AIHA [**1,20,21**]. Treg cells are a subset of T cells, CD4 + CD25hiFoxP3 + and it have a role in maintaining tolerance, protective role in autoimmune diseases but also to initiate oncogenesis [**22**]. The exact involvement of Tregs in CLL is not yet known, however it was shown that malfunction or loss of regulatory T cells (Treg) could have significant involvement in the etiology of autoimmune cytopenia in CLL. It is known that under physiological conditions Treg cells inhibit TH cells, but their decreased number in CLL cause activation of self-reactive TH [**7**]. It has been demonstrated that patients with CLL and AIHA present self-reactive TH cells specific for Rh family antigens (including RhD and RhCcEe epitopes) which are red blood cells (RBC) dominant antigens in AIHA. TH cell self-reactivity could be induced by the pathological presentation of autoantigens mediated by CLL cells [**20,21**].

Some studies showed arguments in favor of T cells participation in the generation of antibodies associated with AIHA and CLL: in patients with idiopathic AIHA - presence of Treg clones specific for Rh epitopes [**22**], Treg cells increase with disease progression in patients with CLL and AIHA compared to CLL patients without AIHA, an inverse relationship between the lymphocyte doubling time and the number of Treg [**23**], the administration of fludarabine, drug considered a risk factor for the development of autoimmune cytopenia in CLL, is followed by decreased Treg cell function [**24**].

e) Abnormalities in the lymphocyte apoptotic program could be an alternative pathogenic mechanism that explains the relationship between lymphocyte proliferation and autoimmune phenomena. The most commonly involved is the Fas / Fas ligand system - important mechanism of cell death. It has an important role in immune response control, peripheral tolerance induction and lymphocyte life span regulation, by elimination of self-reactive lymphocytes during ontogeny [**10,25**]. Moreover, Fas is involved in the TH1 cell cytotoxicity, which is partially due to interaction of FasL expressed by activated cytotoxic cells with Fas expressed by target cells [**26**].

It was identified an autoimmune/ lymphoproliferative syndrome (ALPS) in patients with inherited mutations of the Fas gene, and an ALPS-like clinical pattern (named autoimmune lymphoproliferative disease - ALD) in those with decreased Fas function, but noFas gene mutation. Was suggested that in the same subject, ALD is due to accumulation of several defects and these defects predispose to development of autoimmune diseases and/ or cancer other than ALPS/ALD [**26,27**].

Predisposition to autoimmune diseases in patients with decreased function of the Fas antigen, is multifactorial and may involve factors controlling lymphocyte responsiveness, autoantigen expression and immune effector functions, and may include defects in several levels: in the Fas antigen, in the interaction between Fas and FasLigand or defects of co-stimulatory pathways of B or T cells [**2**].

f) Regarding the etio-pathology of the autoimmune anemia in chronic lymphocytic leukemia B it was observed that the risk of development of AIHA during the evolution of CLL is increased after the administration of some chemotherapeutic agents - alkylating agents, purine analogs and alemtuzumab [**2,28**]. Fludara monotherapy is associated with a 2% risk of developing AIHA after the first administration, the next doses causing a significant increase in risk [**29**]. The mechanism by which the drugs used to treat CLL increase the risk of AIHA is not known, but an explanation would be the toxic effect of this drugs on Treg cells [**2,24**].

In terms of molecular and cytogenetic, the development of AIHA in CLL patients was associated with a specific configuration of B cell receptor (BCR) - HCDR3, unfavorable cytogenetic factors, such as del (17)( p13) and del (11) (q23) and un-mutated genetic status of IgVH (immunoglobulin heavy chain) [**30**]. A different expression of microRNAs (miRNAs) (18-22 nucleotides of RNA molecules) that regulate gene expression in CLL patients with associated AIHA was observed compared to those without this complication [**31**]. About the prognostic factors of CLL, some of them have been associated with autoimmune cytopenias-especially with AIHA: increased number of lymphocytes at diagnosis, peripheral lymphocyte doubling in less than one year, increased beta 2 microglobulin, increased expression of ZAP70, advanced stage disease (Binet "C") [**6**].

## Conclusions

Pathogenic mechanism of autoimmune hemolytic anemia associated with chronic lymphocytic leukemia is very vast, not researched enough, and also far from being completely known. The prognosis of patients with advanced stage of disease and immune anemia is better than the prognosis of patients with advanced disease and anemia secondary to bone marrow infiltration. Generally, in terms of prognosis, AIHA did not demonstrate a significantly influence on prognosis and survival of patients with CLL. In the two most frequently systems used to staging chronic lymphocytic leukemia B: Rai and Binet, the presence of anemia included patients in the high-risk group, assessing survival ~ 30 months. None of these systems does not make a clear tie between cytopenia, depending on their immune or non-immune origin - although they present a clear difference in prognosis (in the intermediate prognosis group- median survival~ 16 months). We believe it is essential to determine the cause of anemia, because it is the only way we can achieve more accurate risk stratification in patients in therapeutic protocols, and also a more effective management of patients who have autoimmune hemolytic anemia associated with CLL.
